# Plant microRNAs: Biogenesis, Homeostasis, and Degradation

**DOI:** 10.3389/fpls.2019.00360

**Published:** 2019-03-27

**Authors:** Junli Wang, Jun Mei, Guodong Ren

**Affiliations:** State Key Laboratory of Genetic Engineering and Ministry of Education Key Laboratory for Biodiversity Science and Ecological Engineering, Institute of Plant Biology, School of Life Sciences, Fudan University, Shanghai, China

**Keywords:** miRNA biogenesis, DCL1, HEN1, Argonaute, target mimic, uridylation

## Abstract

MicroRNAs (miRNAs), a class of endogenous, tiny, non-coding RNAs, are master regulators of gene expression among most eukaryotes. Intracellular miRNA abundance is regulated under multiple levels of control including transcription, processing, RNA modification, RNA-induced silencing complex (RISC) assembly, miRNA-target interaction, and turnover. In this review, we summarize our current understanding of the molecular components and mechanisms that influence miRNA biogenesis, homeostasis, and degradation in plants. We also make comparisons with findings from other organisms where necessary.

## Introduction

RNA silencing, which is mediated by small non-coding RNAs of 20–35 nucleotides in length, is an important and indispensable form of gene regulation among most eukaryotes. According to their origin, processing mode and effector protein association, small RNAs can be divided into four major categories: microRNA (miRNA), small interfering RNA (siRNA), PIWI-interacting RNA (piRNA, animals only), and transfer RNA-derived small RNAs (tsRNAs) (Borges and Martienssen, 2015; Czech et al., 2018; Zhu et al., 2018; Treiber et al., 2019). siRNAs can be further separated into different sub-categories such as heterochromatic siRNAs (hc-siRNAs), phased secondary siRNAs (phasiRNAs) and epigenetically activated siRNAs (easiRNAs) (Borges and Martienssen, 2015; Zhang et al., 2016). Unlike siRNAs and piRNAs, which can mediate gene silencing at either the transcriptional (TGS) or post-transcriptional (PTGS) level, miRNAs predominantly repress target genes post-transcriptionally (Borges and Martienssen, 2015; Bartel, 2018). Plant miRNAs are produced from specific stem regions of single-stranded hairpin precursors, which possess distinct features from other types of small RNAs. Detailed criteria for plant miRNA annotation were described recently (Axtell and Meyers, 2018). If not otherwise specified, we hereinafter refer to plant miRNAs as miRNAs. miRNAs play crucial roles in almost all aspects of normal plant growth and development, but also in response to environmental fluctuations such as light, nutrition, and various abiotic and biotic stresses (Budak et al., 2015; Shriram et al., 2016; Li S. J. et al., 2017; Brant and Budak, 2018). As such, the temporal-spatial expression of intracellular miRNAs is under multi-level control to ensure fine regulation of target genes. Here we review our current understanding of the biogenesis, homeostasis, and turnover of miRNAs, with a focus on the regulation of each step that affects the production or degradation of miRNAs.

## Overview of miRNA Biogenesis, RISC Assembly, and Action

Plant genomes typically encode a hundred to several hundreds of *MIRNA (MIR)* genes, with many of them existing as families (Nozawa et al., 2012; Budak and Akpinar, 2015). According to their location in the genome, miRNAs are classified as either “intergenic” or “intronic.” Intergenic miRNAs are located between two protein-coding genes and are transcribed as independent units by DNA-dependent RNA Polymerase II (Pol II), while intronic miRNAs are processed from introns of their host transcripts (Millar and Waterhouse, 2005; Budak and Akpinar, 2015). As canonical Pol II products, primary transcripts of *MIRs* (termed pri-miRNAs) are 5′ capped, 3′ polyadenylated, and/or spliced (Xie et al., 2005; Rogers and Chen, 2013). pri-miRNAs are folded into hairpin-like structures consisting of a terminal loop, an upper stem, the miRNA/miRNA^∗^ region, a lower stem, and two arms, which can be recognized and processed by Dicer-like RNase III endonucleases (DCLs). Different plant species have different numbers of DCL proteins. In *Arabidopsis thaliana*, there are four DCL proteins. DCL1 catalyzes the production of most miRNAs with the assistance of accessory proteins including the double-stranded RNA-binding protein Hyponastic Leaves 1 (HYL1) and the zinc-finger protein Serrate (SE) (Fang and Spector, 2007; Dong et al., 2008). Other DCLs may also be involved in miRNA production. For instance, AtDCL4 is responsible for miR822 and miR839 production while OsDCL3a in rice generates a class of 24-nt miRNAs that direct DNA methylation like hc-siRNAs (Rajagopalan et al., 2006; Wu et al., 2010). The stem-loops of pri-miRNAs are much more variable in length (from 60 nt to over 500 nt) and bear more complex structures than their ∼70 nt animal counterparts (Xie et al., 2005; Bologna and Voinnet, 2014). As such, pri-miRNAs in plants can be processed from either the loop-distal site to the loop-proximal site or *vice versa* (Addo-Quaye et al., 2009; Bologna et al., 2009, 2013; Mateos et al., 2010; Song et al., 2010; Werner et al., 2010). The nascent miRNA/miRNA^∗^ duplex generated by DCL-mediated processing exhibits 2-nt 3′ overhangs at both strands and each strand possesses a 5′ end phosphate and two 3′ end hydroxyl groups (2′ OH and 3′ OH). While both hydroxyl groups are essential, only the 2′-OH position is methylated by the small RNA methyltransferase HUA Enhancer 1 (HEN1) (Yu et al., 2005; Yang Z. Y. et al., 2006).

**FIGURE 1:**
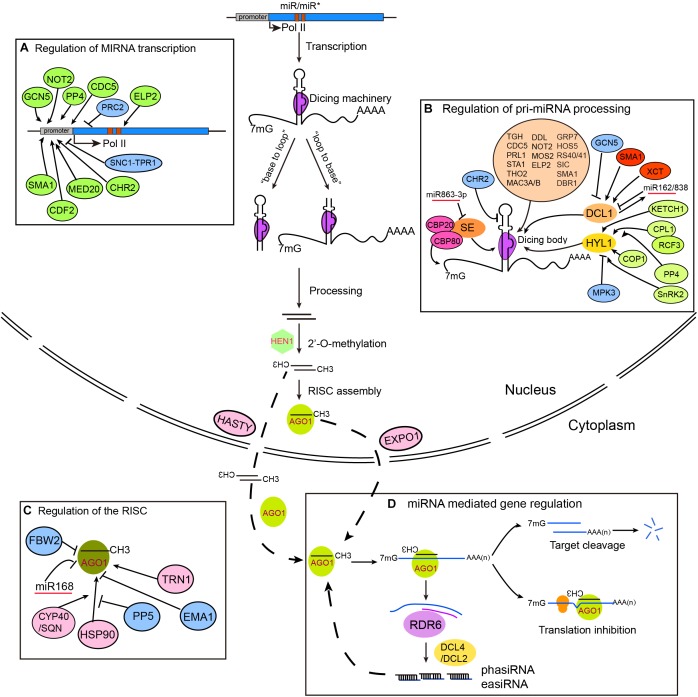
Regulation of miRNA biogenesis, RISC loading, and action in plants. *MIRNA* genes are transcribed by RNA Polymerase II (Pol II) and fold into stem-loop structures called pri-miRNAs. pri-miRNAs are mainly processed by DCL1 from either “base-to-loop” or “loop-to-base” direction. Nascent miRNA/miRNA^∗^ duplexes are methylated by the small RNA methyltransferase HEN1. RISC loading occurs in the nucleus, but may also occur in the cytoplasm. miRNAs mediate gene silencing through either target cleavage or translation inhibition. Some miRNAs can trigger the production of secondary siRNAs through sequential actions of RDR6 and DCL2/4, generating 21–22 nt secondary siRNAs called phasiRNAs and easiRNAs, which in turn repress gene expression *via* PTGS (for phasiRNAs) or TGS (for easiRNAs) **(D)**. It is important to note that although the steps in the model are separate, they could be closely coupled. Factors involved in the regulation of *MIR* transcription, pri-miRNA processing, and RISC assembly are shown in parts **(A–C)**, respectively.

Methylated miRNA/miRNA^∗^ duplexes are thought to be exported by the animal Exportin 5 (EXPO5) homologous protein Hasty (HST) (Park et al., 2005). For a long time, it was not known where the RISC assembled. Recently, Bologna et al. (2018) showed that RISC is mainly assembled in the nucleus and is then exported to the cytosol by EXPO1. However, current data do not exclude the possibility that some miRNAs are exported in their duplex forms and are assembled in the cytosol (Figure 1). One strand of the miRNA/miRNA^∗^ duplex (the guide strand, miRNA) is selectively assembled into the Argonaute (AGO) protein, and the other strand (the passenger strand, miRNA^∗^) is ejected and degraded. Arabidopsis has 10 AGO proteins, with AGO1 being the major effector protein for miRNAs (Zhang H. et al., 2015).

miRNAs guide the RISC to target genes via base pairing and predominantly mediate gene silencing through target cleavage and/or translation inhibition. Nevertheless, recent studies also suggest a role of RISC/AGO1 in transcriptional regulation (Dolata et al., 2016; Liu et al., 2018; Yang et al., 2019). Some miRNAs including miR390, miR173, and miR845 are capable of initiating the production of secondary siRNAs called phasiRNAs and/or easiRNAs (Figure 1D) (Fei et al., 2013; Creasey et al., 2014; Deng et al., 2018). In animals, a short base-pairing to the seed region of miRNAs (positions 2–8) is sufficient for target recognition, although non-canonical targeting has also been observed (Helwak et al., 2013; Agarwal et al., 2015). In contrast, a more stringent base-pairing rule is employed by plants, with near perfect pairing in the 5′ region (no more than 1 mismatch) and relaxed, but ample pairing in the 3′ region (no more than 4 mismatches and only small bulges allowed) (Schwab et al., 2005; Axtell and Meyers, 2018). Theoretically, plants have at least two orders of magnitude fewer target genes than animals. Although translation inhibition seems prevalent, target cleavage is more important as it is essential for post-germination plant development (Carbonell et al., 2012).

## Regulation of *Mirna* Transcription

Similar properties of transcription, co-transcriptional capping, polyadenylation, and splicing of *MIR* genes to coding genes suggest that essentially all known regulatory mechanisms for mRNA transcription may be applied to *MIR* gene transcription (Figure 1A). For instance, changes in the phosphorylation of the Pol II C-terminal domain (CTD) by Cyclin-Dependent Kinase Ds (CDKDs) and CDKF;1 have been reported to modulate *MIR* transcription and co-transcriptional capping, polyadenylation, and splicing (Hajheidari et al., 2012). The transcription co-activator complex mediator plays a general role in recruiting Pol II to *MIR* promoters during transcription initiation (Kim et al., 2011). *MIR* transcription is not only regulated by locus-specific transcription factors and regulators, but is also globally modulated by the CCR4-NOT (for Carbon Catabolite Repression 4-Negative on TATA-less) complex subunit NOT2, the Elongator complex subunits ELP2 and ELP5, the MYB-R2R3 type transcription factor Cell Division Cycle 5 (CDC5), the DOF (for DNA binding with One Finger) transcription factor Cycling DOF Factor 2 (CDF2), the Protein Phosphatase 4 complex, the disease resistance R protein SNC1 (for Suppressor of *npr1-1*, Constitutive 1) and its transcriptional corepressor Topless-Related 1 (TPR1) (Wang et al., 2013, 2019; Zhang et al., 2013; Fang et al., 2015a; Sun et al., 2015; Cai et al., 2018). A general effect of these proteins on *MIR* transcription may be related to their interactions with members of the miRNA processing machinery such as DCL1. The expression levels of *MIRs* are also dynamically regulated by histone modifications (Luo et al., 2013; Zhao et al., 2015). GCN5 (for General Control Non-repressed Protein 5)-mediated H3K14 acetylation promotes the expression of a subset of *MIR* genes, whereas deposition of H3K27me3 by PRC2 (for Polycomb Repressive Complex 2) at *MIR156A* and *MIR156C* genes downregulates their expression and drives the juvenile to adult transition (Kim et al., 2009; Xu et al., 2016).

## Regulation of the Core Processing Machinery

pri-miRNA processing takes place at subnuclear foci called Dicing-bodies (D-bodies) or SmD3/SmB bodies (Fang and Spector, 2007; Fujioka et al., 2007). Among many accessory proteins identified to date, HYL1 and SE are two core cofactors; defects in either HYL1 or SE result in global abolishment of miRNAs and dramatic accumulation of pri-miRNAs (Han et al., 2004; Vazquez et al., 2004; Lobbes et al., 2006; Yang L. et al., 2006). HYL1 and SE may form a complex with DCL1 in D-bodies (i.e., the core processing machinery) to ensure both the precise and efficient cleavage of pri-miRNAs (Fang and Spector, 2007; Dong et al., 2008; Liu et al., 2012; Zhu et al., 2013). The core processing machinery is monitored at multiple levels, including transcriptional, post-transcriptional, and post-translational regulation (Figure 1B).

Transcription of *DCL1* is negatively regulated by the histone acetyltransferase GCN5, and positively regulated by XCT (for XAP5 Circadian Timekeeper) and the pre-mRNA processing factor 6 homolog Stabilized 1 (STA1) (Kim et al., 2009; Ben Chaabane et al., 2013; Fang et al., 2015b). Another splicing factor, SMALL 1 (SMA1), is required for the correct splicing of the ninth intron of *DCL1* (Li et al., 2018). In addition, the abundance of *DCL1* is fine-tuned by two negative feedback mechanisms. First, miR162, a miRNA generated by DCL1, can in turn target *DCL1* mRNA for cleavage. Second, DCL1-mediated processing of *MIR838*, an intronic *MIRNA* gene that resides in the 14th intron of *DCL1* pre-mRNAs, leads to abortive *DCL1* transcription (Xie et al., 2003; Rajagopalan et al., 2006). Indeed, in *hyl1* mutants where such feedback mechanisms are impaired, the abundance of *DCL1* is significantly increased (Liu et al., 2012).

HYL1 functions as a dimer and binds to the stem region of pri-miRNAs (Yang et al., 2010; Yang et al., 2014). The N-terminal double-strand RNA binding domains (dsRBDs) of HYL1 are sufficient for pri-miRNA processing, while the C-terminal part appears dispensable (Wu et al., 2007). Interestingly, expression of the dsRBDs of DCL1 can fully rescue the phenotype of *hyl1* (Liu et al., 2013). Moreover, a suppressor screen identified multiple dominant DCL1 alleles that rescue both the *hyl1* mutant phenotype and miRNA processing defects (Tagami et al., 2009; Liu et al., 2012). These observations indicate an auxiliary, rather than indispensable, role for HYL1 in the recruitment and positioning of pri-miRNAs into DCL1 and facilitating processing. Overexpression of hairpin-like SINE (for Short Interspersed Elements) transposon RNAs can sequester HYL1 from miRNA precursors, leading to reductions in miRNA expression (Pouch-Pelissier et al., 2008). The accumulation of HYL1 is sensitive to light-dark transitions. HYL1 is degraded in the dark by a yet uncharacterized protease in the cytoplasm. While in the light, Constitutive Photomorphogenic 1 (COP1), a RING-finger E3 ligase, can move from the nucleus to the cytoplasm and prevent HYL1 degradation by inhibiting the protease activity (Cho et al., 2014). KETCH1, a member of the importin-β family, is responsible for the translocation of cytoplasmic HYL1 into the nucleus (Zhang et al., 2017b). Post-translational phosphorylation plays a crucial role in regulating both the activity and stability of HYL1. Phosphorylated HYL1 appears non-functional, but can be retained in the nucleus, protecting it from degradation in the dark until it can be reactivated through light-mediated de-phosphorylation (Achkar et al., 2018). Multiple kinases and phosphatases have been characterized in the last few years. Both Mitogen-Activated Protein Kinase 3 (MPK3) and SNF1-related Protein Kinase 2 (SnRK2) were shown to interact with HYL1 *in vivo* and to phosphorylate HYL1 *in vitro* (Raghuram et al., 2015; Yan et al., 2017). On the other hand, C-Terminal Domain Phosphatase-like 1 (CPL1) and the PP4/ Suppressor of MEK 1 (SMEK1) complex were demonstrated to dephosphorylate HYL1 (Manavella et al., 2012; Su et al., 2017). The K homology (KH) domain protein Regulator of CBF Gene Expression 3 (RCF3) promotes HYL1 dephosphorylation through interaction with CPL1 and CPL2 in apex tissues (Karlsson et al., 2015). However, inconsistent results have been observed and await future clarification. For instance, loss of MPK3 results in over-accumulation of HYL1 and increased miRNA levels, whereas the *snrk2* mutant has reduced HYL1 protein levels and decreased miRNA accumulation. Moreover, phosphorylated HYL1 proteins in the *smek1* mutant are unstable and degraded, whereas in another report, phosphorylation protects HYL1 from degradation in the dark by nuclear retention (Raghuram et al., 2015; Su et al., 2017; Yan et al., 2017; Achkar et al., 2018). It is possible that different phosphorylation sites, subcellular localizations, and/or experimental conditions could explain the discrepancy.

Similar to the DCL1-miR162 feedback loop, SE is targeted by miR863-3p. Such regulation occurs during later stages of bacterial infection (Niu et al., 2016). SE is phosphorylated by SnRK2 *in vitro*, although whether this is also the case *in vivo* and what the biological consequence of this modification is remain to be explored (Yan et al., 2017). Interestingly, SE may have dual functions in coordinating miRNA production. First, as a core cofactor of the miRNA processing machinery, SE not only serves as a scaffold protein during microprocessor complex assembly, but may also promote miRNA processing *in vivo*, although a direct role of SE in pri-miRNA processing *in vitro* remains debatable (Dong et al., 2008; Zhu et al., 2013). Second, SE is known to interact with Chromatin Remodeling 2 (CHR2), which remodels pri-miRNAs to inhibit their processing (Wang Z. Y. et al., 2018). It is also worth noting that SE has broader functions than miRNA processing. SE interacts with the cap-binding complex and is involved in pre-mRNA splicing (Laubinger et al., 2008). In a recent study, SE was shown to promote intronless gene expression via direct chromatin binding and facilitating Pol II association (Speth et al., 2018). Moreover, SE is involved in fine-tuning transposon expression *via* promoting H3K27me1 by the Arabidopsis Trithorax Related Protein 5 and 6 (ATXR5/6) and suppressing RNA Dependent RNA Polymerase 6 (RDR6)-mediated RNA silencing (Ma et al., 2018).

## Other Regulatory Proteins Influencing miRNA Biogenesis

A battery of additional regulatory proteins influencing the folding, stability, and/or processing of pri-miRNAs have been identified during the last decade or so, which will not be discussed here (Figure 1B) (for recent reviews, see (Zhang S. X. et al., 2015; Yu et al., 2017b). One of the most important features revealed through these studies is that pri-miRNAs are modified, folded, and processed co-transcriptionally. This is supported by several lines of evidence. First, DCL1 is associated with chromatin regions of *MIR* genes (Fang et al., 2015a). Second, several regulatory proteins affecting miRNA transcription (e.g., CDC5, NOT2, and ELP2) have been shown to interact with processing machinery proteins (Wang et al., 2013; Zhang et al., 2013; Fang et al., 2015a). Notably, a recent study showed that mRNA adenosine methylase (MTA), a homologous protein of animal METTL3, deposits m^6^A onto pri-miRNAs and may impact miRNA biogenesis via its dual interaction with Pol II and Tough (TGH), a known miRNA processing regulator (Ren et al., 2012b; Bhat et al., 2019). Another intriguing finding is that many proteins affecting *MIRNA* gene transcription, pri-miRNA stability, and/or processing have reported or proposed functions in RNA splicing (Yu et al., 2017b).

## Regulation of RISC Assembly and Ago1 Stability

AGO proteins harbor conserved PAZ, MID, and PIWI domains. The MID and PAZ domains bind to the 5′ phosphate and 3′ end of small RNAs, respectively, while the PIWI domain cuts target RNAs through its endonuclease activity (Swarts et al., 2014). The 5′ terminal nucleotide and/or thermodynamic properties of the miRNA/miRNA^∗^ duplex are crucial for AGO sorting and strand selection (Khvorova et al., 2003; Schwarz et al., 2003; Mi et al., 2008; Takeda et al., 2008). AGO1 is the major effector protein and prefers miRNA cargos with a 5′ uracil (Mi et al., 2008). In contrast, AGO7 and AGO10 are predominantly associated with miR390 and miR165/166, respectively (Montgomery et al., 2008; Zhu et al., 2011). The miR390-AGO7 complex is specific for *TAS3* ta-siRNA generation. AGO10 antagonizes AGO1 for miR165/166 loading, which ensures proper development of both shoot and floral apical meristems (Zhu et al., 2011). Interestingly, some miRNA^∗^s can preferentially and stably associate with AGO proteins and function like miRNAs (Mi et al., 2008). For example, miR393b^∗^-AGO2 targets *MEMB12* for translation inhibition during *Pseudomonas syringae* pv. *tomato* infection (Zhang et al., 2011). Cumulative evidence shows that miRNA^∗^s can be as functional as miRNAs, and in some cases, both strands are abundantly expressed. In fact, the miR-5p/miR-3p name format is now adopted by the miRBase database to replace miRNA/miRNA^∗^ (Kozomara and Griffiths-Jones, 2014). Nevertheless, this change brings inconveniences and confusions, because it is almost impossible to know whether a given miR-X-3p refers to miR-X or miR-X^∗^ without checking the annotation.

The assembly of RISC requires the molecular chaperone HSP90, and this process is facilitated by Cyclophilin 40/Squint (CYP40/SQN) and inhibited by Protein Phosphatase 5 (PP5) (Figure 1C) (Iki et al., 2010, 2012; Iwasaki et al., 2010). Loss of CYP40/SQN phenotypically resembles hypomorphic alleles of *ago1*, likely due to reduced miRNA loading efficiency (Smith et al., 2009). CYP40 contains an N-terminal peptidyl-proline isomerase (PPIase) domain and a C-terminal tetratricopeptide repeat (TPR) domain. The TPR domain of CYP40 directly interacts with HSP90 variants containing a C-terminal MEEVD sequence, which is essential for CYP40 function *in vivo* (Earley and Poethig, 2011; Iki et al., 2012). In addition, the RISC loading process is negatively and positively regulated by two importin-β family proteins called Enhanced MiRNA Activity 1 (EMA1) and Transportin 1 (TRN1), respectively (Wang et al., 2011; Cui et al., 2016).

Expression of AGO1 is also under tight regulation. At the post-transcriptional level, *AGO1* transcripts are targeted by miR168. On the other hand, AGO1 loading is important for the stabilization of miRNAs including miR168. Such feedback regulation is crucial for AGO1 homeostasis (Vaucheret et al., 2006). At the post-translational level, the endogenous F-Box protein FBW2 (for F-Box with WD-40 2) targets AGO1 for degradation (Earley et al., 2010). FBW2 was identified and characterized from a forward genetic screen for *sqn* suppressors. FBW2 mutations also suppress weak *ago1* alleles, but not the null *ago1-36* mutant. FBW2 may trigger AGO1 degradation through autophagy rather than the proteasome pathway, because treatment of MG132, a proteasome-specific inhibitor, does not affect AGO1 degradation (Earley et al., 2010). In addition to its roles in miRNA-related RNA degradation, AGO1 is also the major effector in siRNA-mediated post-transcriptional gene silencing and antiviral immunity (Carbonell and Carrington, 2015; Fang and Qi, 2016). AGO1 is one of the major targets of viral silencing suppressors. These include the F-Box Protein P0 of polerovirus and the RNA-binding protein P25 of potato virus X, which target AGO1 for degradation through autophagy and the proteasome pathways, respectively (Baumberger et al., 2007; Bortolamiol et al., 2007; Chiu et al., 2010; Csorba et al., 2010; Derrien et al., 2012).

## miRNA Stability Control

**FIGURE 2:**
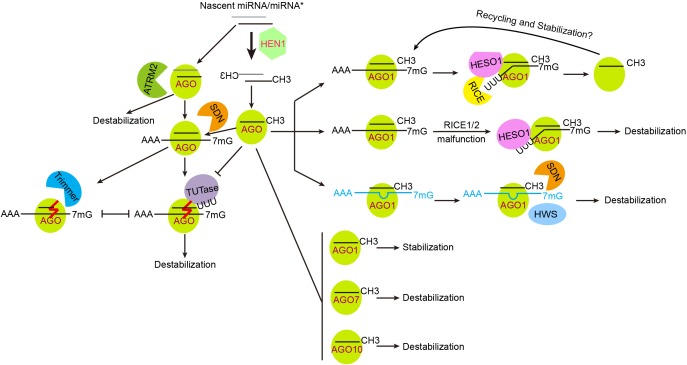
Regulation of miRNA stability and turnover. Plant miRNAs are heavily methylated, which is crucial for their stabilization. ATRM2 is involved in the degradation of unmethylated miRNA/miRNA^∗^s, likely during the initiation of RISC loading. RISC-associated unmethylated miRNAs are destabilized *via* 3′ end tailing and 3′-to-5′ trimming. TUTase, terminal uridylyl transferase such as HESO1 and URT1 in Arabidopsis and MUT68 in *Chlamydomonas reinhardtii.* Trimmer, a yet uncharacterized enzyme catalyzing 3′-to-5′ trimming of unmethylated miRNAs. The effects of AGO proteins on miRNA stability are judged only by the results of *ago* mutants and/or over-expression transgenic plants, and are not necessarily absolute. Defects in the degradation of cleavage products (e.g., in the RICE1/2 malfunction backgrounds) cause miRNA reduction, suggesting that successful release of cleavage products may facilitate RISC recycling and stabilization. TM (in blue color) induced miRNA destabilization involves the actions of SDNs and an F-box protein HWS.

Like all other RNAs, miRNAs have their own half-lives. Turnover and degradation of miRNAs are not only important for maintaining intracellular miRNA homeostasis, but also provide means for their clearance in response to developmental transitions and environmental changes. Factors affecting miRNA stability include 3′ end modification, AGO association, and miRNA-target RNA interaction (Figure 2). In addition, a growing number of exoribonulceases responsible for miRNA degradation at different stages have been characterized.

## Regulation of miRNA Stability by 3′ End Modification

As a self-protection mechanism, mRNAs bear a 5′ cap and a 3′ poly(A) tail after transcription, which ensure their nuclear exportation and promote translation initiation (Garneau et al., 2007). Mature miRNAs lack such structures, but have 2′-*O*-methylation modification at their 3′ end. This modification, as catalyzed by HEN1, represents a critical step in miRNA stabilization in plants (Yu et al., 2005). Little is known about the regulation of HEN1 expression and activity. There is only one report showing that the expression of *HEN1* is relatively low in the dark and is elevated upon light irradiation, which regulates photomorphogenesis by fine-tuning the expression of miR157d and miR319 (Tsai et al., 2014). Loss of HEN1 not only results in disrupted photomorphogenesis and skotomorphogenesis, but also other developmental defects including dwarfism, late flowering, and reduced fertility. At the molecular level, *hen1* mutations cause miRNA destabilization and 3′ end heterogeneity due to combined actions of tailing and 3′-to-5′ truncation. Sequencing analysis of 3′ end tails reveals a dominant addition of uracils, a process that is termed uridylation (Li et al., 2005; Zhao et al., 2012; Wang et al., 2015). In Arabidopsis, HEN1 Suppressor 1 (HESO1) is the major terminal uridylyl transferase that catalyzes the addition of uracils to the 3′ end of unmethylated miRNAs (Ren et al., 2012a; Zhao et al., 2012). Loss of HESO1 greatly suppresses the *hen1* phenotype while overexpression of HESO1 in *hen1* results in further reduced miRNA levels and more drastic developmental defects, demonstrating that uridylation triggers miRNA degradation (Ren et al., 2012a). Another terminal uridylyl transferase, UTP:RNA uridylyltransferase 1 (URT1), only slightly affects the uridylation of a few miRNAs (e.g., miR158) when HESO1 is competent, but can significantly compensate for the loss of HESO1 (Tu et al., 2015; Wang et al., 2015). Different from HEN1, HESO1 and URT1 do not have apparent dsRNA binding domains, arguing against their actions on miRNA/miRNA^∗^ duplexes. Instead, both HESO1 and URT1 colocalize and interact with AGO1, and can uridylate AGO1-bound miRNAs (Ren et al., 2014b; Tu et al., 2015; Wang et al., 2015). Furthermore, miRNA tailing is largely diminished in two hypomorphic *ago1* alleles (Zhai et al., 2013; Ren et al., 2014b). U-tails of unmethylated miRNAs were also abolished in the *heso1-2 urt1-3* mutant, suggesting similar actions of HESO1 and URT1 on miRNAs at molecular level when HEN1 is fully functional (Wang et al., 2015). However, it is not known whether the plant genome encodes any miRNA “demethylases,” because it is difficult to distinguish whether unmetyhlated miRNAs in wild-type plants are demethylated products or those escaped from HEN1 targeting. Small RNA Degrading Nucleases (SDNs, see below for details), which harbors “demethylase”-like function by removing methylated 3′-end nucleotides, cooperate with HESO1/URT1 and contribute to the degradation of some methylated miRNAs (Ramachandran and Chen, 2008; Yu et al., 2017a).

In the unicellular green algae *Chlamydomonas reinhardtii*, the HESO1 homologous protein Mutator 68 (MUT68) uridylates both miRNAs and siRNAs for degradation, implying that uridylation triggered small RNA destabilization is highly conserved (Ibrahim et al., 2010). Interestingly, animal miRNAs do not require protection by methylation, probably due to different complementarity degrees required for target recognition (Ren et al., 2014a,b; Moran et al., 2017). Indeed, incorporation of perfect or near perfect complementary targets results in tailing and trimming of corresponding miRNA in *Drosophila* (Ameres et al., 2010). In addition to uridylation, miRNAs are also subject to oligo-adenylation modification. In *Populus trichocarpa* (black cottonwood), a substantial proportion of miRNAs contain one or more non-template adenines, which play a protective role (Lu et al., 2009). Other types of non-templated nucleotide addition are observed, and become more evident in the *hen1 heso1 urt1* triple mutant, suggesting both hierarchical actions and compensation effects among different terminal nucleotidyl transferases (Wang et al., 2015).

## Exoribonucleases Involved in miRNA Surveillance and Degradation

The requirement of 3′ end methylation for stabilization and 3′-to-5′ truncation of miRNAs in the *hen1* mutant indicates that miRNAs are more likely degraded from 3′ to 5′, although theoretically they could be degraded from both directions by exoribonucleases, or cleaved by endoribonucleases. In *Caenorhabditis elegans*, the 5′-to-3′ exoribonuclease XRN-2 has been implicated in miRNA decay (Chatterjee and Grosshans, 2009). In human HEK293 cells, a subset of miRNAs, including miR382, is degraded by the 3′-to-5′ exosome exoribonucleases RRP41 and XRN-1 (Bail et al., 2010). More recently, the human endonuclease Tudor-SN (TSN) was shown to degrade both AGO2-bound and effector-free miRNAs bearing CA and/or UA dinucleotides (Elbarbary et al., 2017). In addition, a number of 3′-to-5′ exoribonucleases have been reported to target small RNAs in a variety of organisms, such as MUT-7 and Poly(A)-specific Ribonuclease (PARN-1) in *C. elegans*, QDE-2-interacting protein (QIP) in *Neurospora crassa*, Nibbler in *Drosophila*, and PARN-Like Domain Containing 1 (PNLDC1) in silkworms and mice (Tops et al., 2005; Maiti et al., 2007; Han et al., 2011; Liu et al., 2011; Izumi et al., 2016; Tang et al., 2016; Zhang et al., 2017a). The 3′-to-5′ exoribonucleases consist of five superfamilies, DEDD, RRP4, PDX, RBN, and RNR, with each having multiple members in plants (Wang X. Y. et al., 2018). Searching for 3′-to-5′ exoribonucleases involved in miRNA degradation led to the identification of SDNs, which belong to the DEDDh subclass of exoribonucleases (Ramachandran and Chen, 2008). SDN1 is capable of degrading single-stranded small RNAs of 17-27 nt in length *in vitro*, but is inactive on miRNA/miRNA^∗^ duplexes and long single-stranded RNAs. Importantly, methylation only slightly impedes SDN1 activity, making it an excellent candidate for degrading methylated miRNAs (Ramachandran and Chen, 2008). Deep sequencing analysis of AGO1- and AGO10-bound miRNAs after SDN1 treatment suggests that SDN1 is responsible for the truncation of both methylated and unmethylated miRNAs, which are further degraded via uridylation-dependent or -independent pathways (Yu et al., 2017a; Chen et al., 2018). Structural and biochemical analysis of SDN1 further reveals a dynamic interaction among SDN1, AGO1 and target RNA, which provides detailed insights into the action of SDN1 on AGO-bound miRNAs (Chen et al., 2018). Since SDN1 is unresponsive to miRNAs with U-tails *in vitro*, the nuclease that degrades uridylated miRNAs remains to be identified. In animals, the RNase II family exoribonuclease DIS3L2 specifically binds and degrades uridylated RNA substrates including uridylated pre-let-7, 7SL, and snRNAs (Chang et al., 2013; Labno et al., 2016; Pirouz et al., 2016). DIS3L2 belongs to the RNR superfamily and the closest homologous gene to DIS3L2 in Arabidopsis is SOV. However, SOV is inactive in the Col-0 background, reducing its likelihood of being the enzyme that degrades uridylated miRNAs (Zhang et al., 2010). In *Chlamydomonas*, the exosome auxiliary nuclease RRP6 acts cooperatively with MUT68 in the degradation of unmethylated miRNAs (Ibrahim et al., 2010). There are three RRP6 homologous proteins with different subcellular locations in Arabidopsis. However, the quadruple mutant *hen1 rrp6l1 rrp6l2 rrp6l3* is morphologically indistinguishable from the *hen1* single mutant, suggesting that RRP6-like proteins are unlikely involved in degrading uridylated miRNAs (Wang X. Y. et al., 2018). Interestingly, knock-out of ATRM2, a DEDDy type exoribonucleases, significantly rescues the *hen1* phenotype. ATRM2 acts downstream of HEN1 and may be involved in the degradation of unmethylated miRNA/miRNA^∗^ duplexes during RISC assembly (Figure 2) (Wang X. Y. et al., 2018).

## Ago Proteins Affecting miRNA Stability

AGO proteins not only serve as the effector protein, but also influence the stability of miRNAs. Arabidopsis AGO1 is suggested to stabilize miRNAs in general since miRNA abundances are decreased in the *ago1* knock-out mutant (Vaucheret et al., 2004). Similar observations are obtained in human cells, where overexpression of AGO2 promotes miRNA accumulation and increases the half-lives of some miRNAs, while knocking out AGO2 reduces miRNA levels (Diederichs and Haber, 2007). The effect of AGO2 on miRNA stability appears independent of its endonuclease activity as miRNA abundances are not affected in an *ago2* mutant specifically defective in cleavage (Diederichs and Haber, 2007). However, different AGO proteins may have different effects on the stability of their bound miRNAs (Figure 2). For instance, Arabidopsis *ago10* mutants exhibit over-accumulation of miR165/166, whereas overexpression of AGO10 results in miR165/166 reduction (Yu et al., 2017a). Similarly, in both tomato and Arabidopsis *ago7* mutants, increases in miR390 abundance are observed, although the underlying mechanism is unknown (Yifhar et al., 2012; Li J. et al., 2017). As multiple degradative enzymes, such as SDN1, HESO1, and URT1, act on AGO-associated miRNAs, it is not surprising that AGO proteins may simultaneously have two opposing roles on miRNA stability (i.e., protect miRNAs from exposure to various intracellular RNases on the one hand, while recruiting degradative factors for active turnover on the other hand).

## Target Rnas Affecting miRNA Stability

As mentioned above, introduction of highly complementary target RNAs initiates miRNA destabilization in animals *via* tailing and truncation at their 3′ ends, mimicking results observed in plant *hen1* mutants (Li et al., 2005; Ameres et al., 2010). The phenomenon is termed target RNA-directed miRNA degradation (TDMD) in animals (de la Mata et al., 2015). This mutual degradation mechanism (i.e., between a miRNA and its target genes), together with a miRNA gradient generated by cell-to-cell diffusion, sharpens spatial expression of target genes, which plays a critical role in morphogenesis such as root vascular patterning and leaf polarity establishment (Hamelryck et al., 2006; Muraro et al., 2014; Ramachandran et al., 2017). In addition to highly complementary target RNAs, mimic targets can also trigger destabilization of miRNAs (Figure 2). Target mimicry was initially reported in plants (Franco-Zorrilla et al., 2007). A non-coding gene, *IPS1*, harbors a non-canonical miR399 targeting site by the presence of a 3-nt bulge at the miR399 cleavage site, which inhibits its cleavage by miR399. *IPS1* serves as a target mimic (TM) to sequester miR399 from its endogenous true target genes (Franco-Zorrilla et al., 2007). Target mimicry may be widespread since many potential endogenous target mimics (eTM) were found in the Arabidopsis, rice and wild emmer wheat genomes based on bioinformatic predictions (Wu et al., 2013; Akpinar et al., 2018). MIM and Short Tandem Target Mimic (STTM) are artificially designed TM technologies. Both TM strategies effectively reduce miRNA abundance (Todesco et al., 2010; Yan et al., 2012). SDN1/2 is involved in this process, as the *sdn1 sdn2* double knock-out mutant significantly suppresses developmental defects caused by STTM (Yan et al., 2012). It is probable that TM may lead to conformational and/or post-translational modification changes in AGO1 (Golden et al., 2017; Huberdeau et al., 2017; Kobayashi et al., 2019), which releases the 3′ end of miRNAs from the PAZ domain of AGO1 and increases their susceptibility to SDN1/2 (Chen et al., 2018). Moreover, loss of function in an F-box family protein HWS (Hawaiian Skirt) suppresses both MIM and STTM-induced developmental defects (Lang et al., 2018; Mei et al., 2018). Intriguingly, miRNA and its corresponding mimicry targets are stably coexisted in the AGO1 immunoprecipitates when HWS is compromised, suggesting that HWS may specifically trigger degradation of non-optimal RISCs (RISCs associated with mimicry target RNAs) (Mei et al., 2018) (Figure 2). The relationship between SDN1/2 and HWS in TM-induced miRNA decay awaits future investigation. Although targets with high complementary cause miRNA destabilization, mRNA targeting could be beneficial for miRNA stabilization (Figure 2). In *C. elegans*, efficient targeting protects let-7 miRNA from XRN2-mediated 5′-to-3′ clearance (Chatterjee and Grosshans, 2009). In Arabidopsis, DnaQ-like exonucleases RICE1 and 2 (for RISC-interacting Clearing 3′-to-5′ exoribonucleases) interact with AGO1 and AGO10, and degrade uridylated miRNA cleavage products for their clearance (Ren et al., 2014b; Zhang et al., 2017c; Zuber et al., 2018). Disruptions of RICE function by either simultaneously knocking down RICE1 and 2 or overexpressing catalytically inactive RICE1 leads to reduced miRNA levels. These data suggest that over-accumulation of cleaved targets could also affect the stability of RISC-associated miRNAs (Zhang et al., 2017c).

## Future Perspectives

The identification of many players in miRNA transcription and processing has shed light on co-transcriptional splicing, modification, and processing of pri-miRNAs. However, key challenges remain with respect to the relationships and precise biochemical contributions of these players. At the subcellular level, it is of particular interest to investigate how the dicing body is formed, its composition, and its role in co-transcriptional pri-miRNA processing. Implementation of novel techniques such as single-cell biology and *in vitro* re-constitution of the dicing machinery will be crucial to tackle these problems. Although many factors, such as AGOs, targets, and 3′ modifications are known to affect miRNA stability, not much is known about the underlying mechanisms. Future studies are needed to identify and characterize additional enzymes and modulators involved in these processes. It will also be important to understand the biological significance of either global or sequence-specific miRNA degradation during developmental transitions and in response to environmental stimuli.

## Author Contributions

JW and JM drafted the manuscript and the figures. GR conceived the idea and revised the manuscript.

## Conflict of Interest Statement

The authors declare that the research was conducted in the absence of any commercial or financial relationships that could be construed as a potential conflict of interest.
